# Uncertainty-Aware Prediction Across Endoscopic Domains: Laryngeal Narrow-Band and Gastrointestinal Imaging

**DOI:** 10.3390/biomedicines14071616

**Published:** 2026-07-17

**Authors:** Behnam Kiani Kalejahi, Sajid Khan, Murodbek Akhrorov, Mohammad Javad Rajabi, Ahmed Aziz

**Affiliations:** 1 Department of Computer Science, School of Applied Intelligence, Central Asian University, Tashkent 111211, Uzbekistan; 2 Faculty of Data Science and Information Technology, INTI International University, Nilai 71800, Malaysia; 3 Medical School, Central Asian University, Tashkent 111221, Uzbekistan; 4 Computer Science Department, Faculty of Computer and Artificial Intelligence, Benha University, Banha 13511, Egypt

**Keywords:** narrow-band imaging, contact endoscopy, domain shift, uncertainty quantification, selective prediction, deep ensembles, laryngeal cancer, gastrointestinal endoscopy, calibration, human health

## Abstract

**Background**: Deep-learning systems for endoscopic image classification are commonly evaluated with random data splits, which may overestimate performance under acquisition shift; uncertainty-aware selective prediction may improve reliability by allowing a model to abstain on uncertain cases. **Methods**: We evaluated binary abnormality detection in two endoscopic imaging domains: laryngeal contact-endoscopy narrow-band imaging (CE-NBI; 210 patients, patient-level) and gastrointestinal endoscopy (HyperKvasir; 6746 images, image-level, predominantly white-light). ImageNet-pretrained ResNet-50 deep ensembles were assessed under a resolution-defined acquisition-shift stress test; for the gastrointestinal data, a random stratified split was additionally used as an in-distribution reference. We evaluated discrimination, calibration, decision-curve analysis, and entropy-based selective prediction. **Results**: In the gastrointestinal dataset, random-split evaluation produced high performance (AUROC 0.994, 95% CI 0.990–0.996; AUPRC 0.991). Under resolution shift on the same data, performance fell to AUROC 0.723 (95% CI 0.710–0.736; AUPRC 0.690; sensitivity 0.417; specificity 0.874); the two intervals do not overlap. Selective prediction improved reliability among retained cases: under resolution shift, accuracy rose from 0.683 at full coverage to 0.852 (95% CI 0.835–0.871) at 25% coverage (balanced accuracy 0.646 → 0.798). Predictive entropy was significantly higher for incorrect than for correct predictions in both regimes (Mann–Whitney *p* = 7.1 × 10^−77^ with rank-biserial |r| = 0.30 under shift). In the laryngeal cohort, no statistically significant differences were detected among four architectures (ROC-AUC 0.844–0.901; all pairwise DeLong *p* > 0.05). **Conclusions**: Random-split evaluation substantially overestimated performance relative to a resolution-defined acquisition-shift stress test, and entropy-based selective prediction improved reliability by identifying a high-confidence subset for automated prediction while deferring the remainder to human review. Target-domain recalibration substantially restores calibration under shift (ECE 0.172 → 0.036 with temperature scaling; → 0.017 with isotonic regression) but does not recover discrimination; selective prediction is complementary, mitigating residual confident-wrong predictions. An encoder-transfer experiment showed asymmetric cross-domain utility; features learned on the larger gastrointestinal cohort transferred to the laryngeal cohort (AUROC 0.80 vs. in-domain 0.89), whereas the reverse direction did not transfer (0.53 vs. 0.72). Prospective multi-center validation remains required before clinical deployment. All code, fold definitions, random seeds, and a reproducible protocol are publicly released.

## 1. Introduction

Narrow-band imaging (NBI) enhances visualization of mucosal microvasculature and improves detection of premalignant and malignant lesions across endoscopic specialties, from the larynx to the gastrointestinal (GI) tract [1,2,3,4,5]. Deep learning has shown strong performance for NBI image classification, but most studies share three weaknesses that limit clinical translation: evaluation under random data splits that share acquisition characteristics between training and test; reporting confined to discrimination metrics; and the absence of any mechanism for the model to signal when its prediction should not be trusted [6,7,8,9,10,11].

Acquisition domain shift, variation in image resolution, optics, and processing across devices, sites, and time, is the rule rather than the exception in deployment. A model that excels on a random split of one center’s data may degrade sharply on images acquired differently. Equally, a screening adjunct that cannot abstain on cases it is unsure about offers little safety margin. These concerns are organ-agnostic, which raises a question rarely addressed: do the conclusions one draws under domain shift generalize across endoscopic domains, or are they artifacts of a single small cohort?

We address this directly by studying the same problem, binary malignancy/abnormality detection under an explicit resolution-shift protocol, in two very different NBI settings: laryngeal CE-NBI (a small, patient-level cohort) and GI endoscopy (a large, image-level cohort). This pairing lets the small clinical cohort supply real-world motivation, while the large cohort supplies statistical power, and lets us test whether an uncertainty-aware selective-prediction method transfers across organs.

### 1.1. Related Work

Deep learning for NBI lesion classification. For laryngeal CE-NBI, Esmaeili et al. characterized vascular patterns and trained CNNs to separate benign from malignant vocal-fold lesions, reporting test accuracy of approximately 0.84 with a fine-tuned ResNet-50 on a random image-level split [3,4,5,11]. For GI endoscopy, the HyperKvasir benchmark reported strong multi-class performance with averaged ResNet-152/DenseNet-161 ensembles [12], and subsequent studies have reported high accuracy (in the ~0.95–0.97 range) with DenseNet variants, again on random splits [7]. These results establish that NBI lesion classification is highly tractable in-distribution; none of these studies, however, evaluates under an explicit acquisition-resolution shift, and none reports an abstention mechanism.

Domain shift and uncertainty in medical imaging. Dataset shift is a recognized threat to medical AI generalization [10,13]. Deep ensembles provide simple, well-calibrated predictive uncertainty [14]; selective classification formalizes the accuracy–coverage trade-off when a model may abstain [15]; and post hoc temperature scaling improves calibration of modern networks [16]. These tools are mature in the machine-learning literature but are rarely transferred into, and almost never validated across, clinical endoscopic domains under controlled distribution shift.

Gap addressed. To our knowledge, no prior work (i) quantifies, on identical data, how much random-split evaluation overstates NBI performance relative to acquisition-shift evaluation, nor (ii) demonstrates, to our knowledge for the first time, that an uncertainty-aware selective-prediction mechanism transfers across two distinct endoscopic domains under such shift. We provide evidence addressing this gap.

### 1.2. Contributions

1. A unified acquisition-resolution domain-shift evaluation protocol for NBI, applied identically across two anatomical domains (larynx, GI) and contrasted directly with conventional random-split evaluation, is, to our knowledge, the first such cross-domain protocol in this setting.
2. A large-sample, same-data quantification (n = 5909 GI test images) showing random-split evaluation overstates ROC-AUC by 0.27 (0.994 → 0.723) relative to domain-shift evaluation, evidence that the optimistic numbers common in the NBI literature are partly an artifact of evaluation design.
3. An honest multi-architecture benchmark (laryngeal) showing that, under domain shift on a small cohort, architecture choice does not significantly change discrimination.
4. The principal contribution: an uncertainty-aware selective-prediction framework (deep-ensemble predictive entropy) shown to improve reliability in both organs and under both regimes, is, to our knowledge, the first demonstration that the abstention remedy transfers across these two endoscopic domains, with calibration and decision-curve analyses absent from prior NBI work.
5. Full release of code, fold definitions, and a reproducible protocol, plus a documented evaluation pitfall (an untrained-head baseline) for the community.

## 2. Materials and Methods

### 2.1. Datasets

Laryngeal CE-NBI. The public CE-NBI cohort [14] comprises 210 patients and 11,144 NBI frames (96 dpi JPEG of varying dimensions). Patient-level labels were malignant (squamous cell carcinoma) versus benign. Evaluation is patient-level via multi-instance aggregation.

Gastrointestinal endoscopy (HyperKvasir). The public HyperKvasir dataset [12] provides 10,662 labeled images across anatomical, pathological, therapeutic, and mucosal-quality categories (from a larger collection of 110,079 images). We defined a binary task of pathological findings (polyps, esophagitis, ulcerative colitis, Barrett’s; n = 2642) versus normal anatomical landmarks (n = 4104), excluding therapeutic and mucosal-quality categories as clinically ambiguous, yielding 6746 images (pathological prevalence 39.2%). HyperKvasir images are not patient-grouped, so this domain is evaluated at the image level.

**Figure 1:**
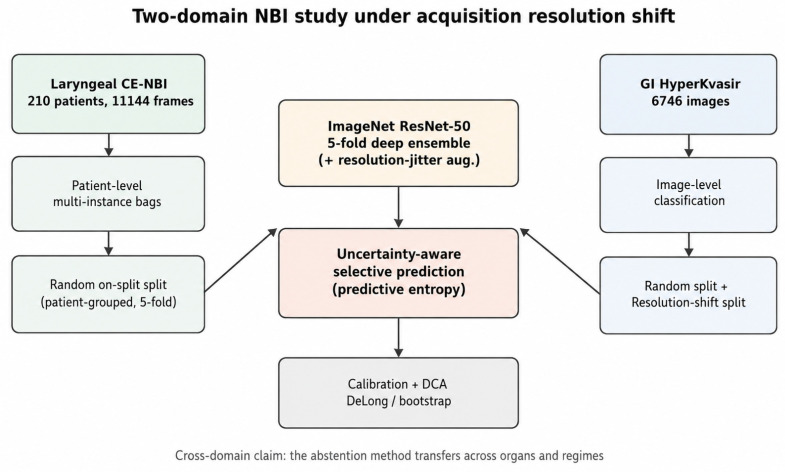
Study design. Two anatomically distinct NBI domains, laryngeal CE-NBI (patient-level) and gastrointestinal HyperKvasir (image-level), are evaluated under a common acquisition-resolution domain-shift protocol with a shared ImageNet ResNet-50 deep-ensemble pipeline, uncertainty-aware selective prediction, calibration, and decision-curve analysis.

Class-to-label mapping (HyperKvasir). Positive class (n = 2642): polyps (1028); ulcerative-colitis grades 0–1 (35), 1 (201), 1–2 (11), 2 (443), 2–3 (28), and 3 (133); esophagitis A (403) and esophagitis B–D (260); Barrett’s (41) and Barrett’s short-segment (53); and hemorrhoids (6). Negative class (n = 4104): cecum (1009), pylorus (999), z-line (932), retroflex-stomach (764), retroflex-rectum (391), and ileum (9). Excluded as clinically ambiguous: therapeutic interventions (dyed-lifted-polyps 1002, dyed-resection-margins 989) and mucosal-quality categories (bbps-2–3 1148, bbps-0–1 646, impacted-stool 131) (Figure 1).

### 2.2. Resolution-Shift and Random-Split Protocols

In both domains, the primary protocol is a resolution-shift split: the majority image-resolution group forms the training/validation pool and the remaining resolutions form the held-out test set, so that test images are acquired under a different resolution profile than training. For the GI domain, we additionally report a random stratified split (class-balanced, same distribution in train and test) as an in-distribution reference, enabling a direct same-data contrast between random-split and domain-shift evaluation. For the laryngeal domain, splitting is strictly patient-level with five-fold StratifiedGroupKFold (scikit-learn StratifiedGroupKFold, random_state = 42), with Patient ID as the group key so no patient appears in more than one fold; per-fold counts of benign/malignant patients are included in the released outputs to verify class balance within the training pool. We emphasize that the resolution-shift split is a reproducible acquisition-shift stress test, not a substitute for multi-center external validation: image resolution may be partly confounded with anatomy, pathology subtype, preprocessing, or device, and the resulting train/validation pool for the GI shift experiment is deliberately small (837 images) relative to the test set (5909), so the observed degradation reflects the combined effect of these acquisition-correlated factors rather than resolution alone.

Formalism. Let X denote endoscopic images and Y the binary label. Random-split evaluation assumes train and test images are drawn from the same joint distribution P(X, Y). The resolution-shift protocol instead assumes a covariate shift: the marginal P_shift_(X) differs from P_in_(X) by construction; training images come from the majority resolution group and test images from the remaining resolutions, while the conditional label distribution P(Y|X) is assumed to be invariant. The proposed selective-prediction mechanism relies on this conditional-invariance assumption: predictive entropy is informative about correctness only insofar as the same image semantics carry the same labels in train and test. If concept drift were also present (P(Y|X) changing across the resolution groups), our framework would not separately identify it; we treat this as a known limitation of the protocol.

Resolution definitions. For the gastrointestinal data, the majority resolution group, defined by the modal pixel dimensions across the labeled-image set, is 1349 × 1071 pixels (837 images forming the train/validation pool); test images come from all other native resolutions in HyperKvasir (5909 images). All images are bilinearly resized to 224 × 224 before encoding. The shift is therefore a true multi-resolution stress test (the test images were acquired with native dimensions different from the training majority), not a controlled downsample-then-upsample of a single source. The laryngeal CE-NBI images are 96 dpi JPEGs of varying native dimensions; the same protocol partitions patients by their majority frame-resolution group.

### 2.3. Models and Training

We selected ImageNet-pretrained ResNet-50 [17] as the shared backbone because it is the de facto reference architecture in published CE-NBI [3,4,5,11] and HyperKvasir [12] benchmarks, enabling direct comparison with prior work; the contribution of this study is methodological (evaluation protocol + abstention framework) rather than architectural, so a widely used baseline was preferred over a newer architecture that might confound interpretation. Larger or more recent encoders (Vision Transformers, ConvNeXt, EfficientNet) are a natural extension and are noted in Future Directions [18]. The model is fine-tuned end-to-end, with images resized to 224 × 224 and converted to tensors (no channel normalization), and training-time augmentation including random resized crop, horizontal flip, rotation, color jitter, and a resolution-jitter augmentation that randomizes effective resolution. Optimization used AdamW (learning rate 5 × 10^−5^, weight decay 1 × 10^−4^) with cosine annealing and early stopping on validation ROC-AUC. Each domain was modeled as a five-fold deep ensemble; the ensemble mean is the prediction and the spread across folds, together with predictive entropy, provides uncertainty. The laryngeal domain additionally compared a single-frame model, a multi-task model with an auxiliary leukoplakia head, and a gated dual-branch multi-instance learning (MIL) model; the GI domain used the single-frame ResNet-50 ensemble.

Ensemble diversity. The five GI ensemble members produced non-trivial diversity (mean pairwise probability correlation 0.79, minimum 0.75; mean pairwise binarized-prediction disagreement rate 0.21; Fleiss’ κ = 0.557, indicating moderate agreement). This confirms that entropy across the ensemble carries genuine epistemic information rather than collapsing to a single-model softmax confidence; the abstention performance therefore reflects ensemble-derived uncertainty, not a single network’s overconfidence.

Disentangling the contributions of ensembling and entropy-based abstention. Two mechanisms could each plausibly explain the selective-prediction gain: (a) ensembling itself averages out per-model errors, and (b) the entropy signal computed across ensemble members ranks cases by reliability. The released analyses show both contribute but in distinguishable ways. The diversity metrics (mean pairwise probability correlation 0.79, mean disagreement 0.21, Fleiss’ κ 0.557—moderate agreement) confirm the five ensemble members are non-redundant: a near-degenerate ensemble (κ → 1) would collapse predictive entropy to a single-model softmax confidence, and Table 1 shows ensemble entropy (AUACC 0.786) outperforms single-model softmax confidence (AUACC 0.779) by a small but consistent margin under shift—the ‘ensembling per se’ contribution. The larger effect is the abstention mechanism itself: at 25% coverage, accuracy rises from 0.683 to 0.852 on the retained subset, an improvement no individual signal in Table 1 achieves at full coverage. Operationally, ensembling produces a diverse probability set whose dispersion (entropy/variance) is a usable per-case reliability score; the abstention rule then realizes the gain. We therefore frame the contributions as multiplicative rather than additive: ensemble diversity is a precondition for entropy to be informative, and the selective-prediction rule is what converts that informativeness into clinical reliability on the retained subset.

Laryngeal multi-instance aggregation. Each laryngeal patient is represented as a bag of CE-NBI frames. Frame features from the ResNet-50 encoder are aggregated by attention pooling [19]; the gated dual-branch variant maintains two attention branches (general and leukoplakia-specialized) combined through a learnable temperature-controlled gate, with an auxiliary leukoplakia-classification head supervised by the public per-patient leukoplakia flag. Patient-level test predictions average K = 10 randomly sampled bags with horizontal-flip test-time augmentation, reducing sampling variance. Bag sizes were phenotype-conditioned (24 frames for non-leukoplakic, 40 for leukoplakic patients).

Gated dual-branch MIL architecture (exact specification). The encoder produces 2048-dimensional features per frame (h_i_ ∈ ℝ^2048^). Each attention-pooling branch computes attention weights a_i_ = softmax_i_(w·tanh(V h_i_)), where V ∈ ℝ^(256×2048)^ and w ∈ ℝ^(256×1)^ are learnable; the softmax is taken over instances within a bag, so weights sum to 1. The bag representation of each branch is z = Σ_i_·a_i_·h_i_ ∈ ℝ^2048^. The two branches (general and leukoplakia-specialized) are mixed by a learnable gate g(z_general_, z_leuko_) = σ((W_g_ [z_general_; z_leuko_] + b_g_)/τ) with W_g_ ∈ ℝ^(1×4096)^, τ = 2.0; the bag-level logit is (1 − g) logit_general_ + g logit_leuko_. An auxiliary leukoplakia head supervised by the public leukoplakia flag (λ_aux_ = 0.30) and a gate-entropy regularizer (λ_ent_ = 0.02, gate frozen at 0.5 for two warm-up epochs) complete the architecture. The released code contains the full module definition. 

Ensembling and uncertainty. For each domain, five models were trained on the cross-validation folds and combined by probability averaging. The ensemble both improves robustness and yields two complementary uncertainty signals: the predictive entropy of the mean probability (total uncertainty) and the variance of the per-fold probabilities (epistemic disagreement). Unless stated otherwise, abstention uses predictive entropy. Training used mixed-precision arithmetic; the random seed was fixed (43) and cross-validation folds were generated with a fixed seed (42) to support exact reproduction. All experiments were implemented in Python (3.8+) with PyTorch, torchvision, scikit-learn, and NumPy; the full code is openly available in the repository listed in the Data Availability Statement.

Resolution-jitter augmentation (formal specification). With probability 0.8 per training image, we apply f(x) = Ψ(Ψ_s_(x)), where Ψ_s_ downsamples bilinearly by factor s ∼ Uniform(0.35, 1.0) and Ψ upsamples bilinearly back to the original size; with conditional probability 0.3, a Gaussian blur with σ ∼ Uniform(0.3, 1.2) is then applied. Augmentation is sampled independently per image (and therefore per ensemble member through their respective mini-batches). The evaluation transform (resize to 224 × 224 + tensor conversion, no channel normalization) is unchanged at test time.

### 2.4. Selective Prediction, Calibration, and Statistics

Selective prediction ranked cases by predictive entropy and progressively abstained on the most uncertain, measuring accuracy, balanced accuracy, and ROC-AUC on the retained subset versus coverage; the area under the accuracy–coverage curve summarizes the trade-off. Calibration used expected calibration error (ECE; B = 15 equal-width bins, |B|-weighted), the Brier score, the negative log-likelihood (NLL), and the maximum calibration error (MCE), with post hoc temperature scaling. Discrimination differences (laryngeal) were tested by DeLong and the paired bootstrap. Predictive entropy of correct versus incorrect cases was compared by the Mann–Whitney U test.

Formally, for an input *x*, the five folds produce probabilities whose mean is the ensemble prediction p(x). The binary predictive entropy is:

*H*(*x*) = − *p log*_2_ *p* − (1 − *p*) *log*_2_(1 − *p*).

Given coverage c ∈ (0,1], the selective predictor retains the fraction c of cases with the lowest H(x) and abstains on the rest; we report accuracy and balanced accuracy on the retained subset versus c, and summarize the trade-off by the area under the accuracy–coverage curve (AUACC). Calibration is measured by expected calibration error over M = 15 equal-width confidence bins.

For the deep ensemble used in this study (M = 5 members), the ensemble predictive probability is the arithmetic mean of per-member softmax outputs, p(c|x) = (1/M) Σ_m_ p_m(c|x), and the ensemble predictive entropy is H(ỹ|x) = −Σ_c_ p(c|x)·log p(c|x) (total predictive uncertainty). Temperature scaling, when applied, divides the ensemble logits by a single scalar T fitted on a held-out calibration partition after ensemble averaging, so it does not change the rank order of cases by entropy and therefore does not affect coverage thresholds. Predictive entropy was chosen as the primary uncertainty score because it is the standard summary of total predictive uncertainty for deep ensembles [14]; alternative signals (single-model softmax confidence, ensemble variance) are evaluated as baselines in Table 1, and Monte-Carlo dropout, evidential, and conformal approaches are noted as future work.

*ECE* = *Σ_m_* (|*B_m_*|/*N*)| *acc*(*B_m_*) − *conf*(*B_m_*) |,

and by the Brier score; post hoc temperature scaling divides the logits by a scalar T fitted on a held-out calibration partition. The ΔAUC between laryngeal models was tested with DeLong [20] and a 5000-sample paired bootstrap. All laryngeal AUROC, DeLong, and bootstrap analyses use n = 73 patient-level scores (multi-bag predictions averaged to a single score per patient) as the unit of analysis, not individual frames; resulting CI half-widths (≈0.07) reflect this limited patient count. Ninety-five percent confidence intervals for GI metrics used a non-parametric bootstrap (B = 2000) stratified by class label to preserve prevalence, the percentile method. Because HyperKvasir lacks patient grouping, GI resampling is at the image level, which does not account for any within-examination correlation and may yield slightly optimistic interval widths; this is stated as a limitation.

### 2.5. A Documented Evaluation Pitfall

During this work, we identified that a baseline built by loading only encoder weights into a network with a randomly initialized, untrained classifier head yields near-random discrimination (ROC-AUC ≈ 0.58) and can manufacture spuriously large apparent gains. We excluded such baselines and verified that every comparator’s classifier was trained. We report this as a caution for the field.

## 3. Results

### 3.1. Random-Split Evaluation Overstates Performance (Gastrointestinal)

**Figure 2:**
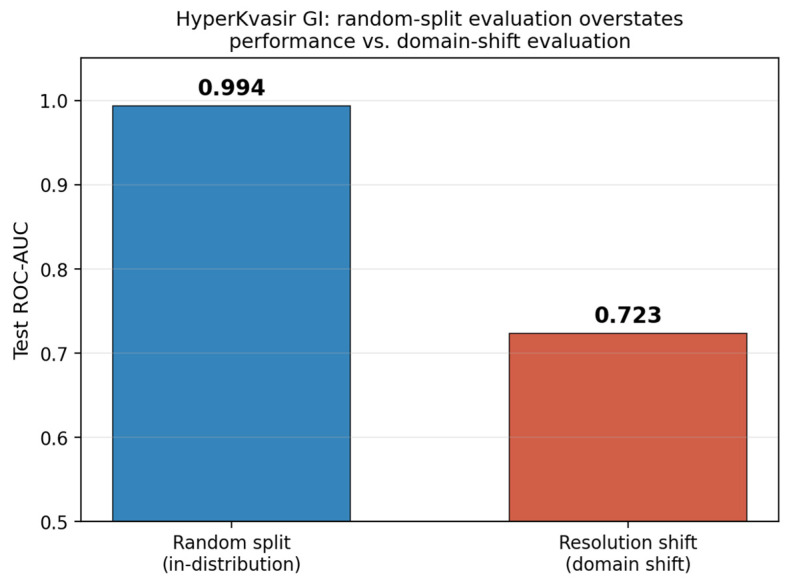
Same GI data, two evaluation protocols. Random-split (in-distribution) ROC-AUC 0.994 versus resolution-shift (domain-shift) ROC-AUC 0.723. Random-split evaluation overstates deployable performance by 0.27 AUC.

On the GI data, the five-fold ResNet-50 ensemble achieved excellent in-distribution performance under a random stratified split (test n = 1350): ROC-AUC 0.994 (95% CI 0.990–0.996), PR-AUC 0.991, accuracy 0.966, balanced accuracy 0.962, sensitivity 0.945, and specificity 0.979. On the identical data under the resolution-shift split (test n = 5909), performance fell to ROC-AUC 0.723 (95% CI 0.710–0.736), PR-AUC 0.690, accuracy 0.683, and balanced accuracy 0.646, with high specificity (0.874) but low sensitivity (0.417); the model became conservative and missed pathology under shift (Figure 2, Table 2). This 0.27 AUC gap, on a large test set, is a direct demonstration that random-split evaluation substantially overstates the performance a deployed model would achieve; the two 95% confidence intervals (0.990–0.996 vs. 0.710–0.736) do not overlap, so the gap is statistically unambiguous rather than a sampling artifact.

### 3.2. Architecture Has Limited Effect Under Domain Shift (Laryngeal)

On the laryngeal resolution-shift test set (n = 73 patients), a single-frame ResNet-50, a multi-task model, an averaged ensemble of the two, and a gated dual-branch MIL achieved ROC-AUC of 0.887, 0.895, 0.901, and 0.844 respectively. No pairwise difference reached significance (all six DeLong *p* = 0.090–0.659; smallest Gated MIL vs. SF+MT ensemble *p* = 0.090; bootstrap ΔAUC intervals all crossed zero). On this cohort, the architectures are statistically indistinguishable: in this small test set, no significant differences were detected between them; this suggests that architectural gains may be limited or difficult to demonstrate under domain shift without substantially larger cohorts, rather than proving exact equivalence.

Notably, at the Youden-optimal operating point, all four laryngeal models achieved 100% sensitivity on the 23 malignant patients and differed only in specificity (0.60–0.74), with the SF+MT ensemble being most specific. In a malignancy-screening context, this error profile, no missed cancers and some benign lesions flagged for confirmation, is the clinically preferable direction, though the small malignant count (n = 23) warrants caution in interpreting the perfect sensitivity.

### 3.3. Uncertainty-Aware Selective Prediction Generalizes Across Organs (Principal Result)

**Figure 3:**
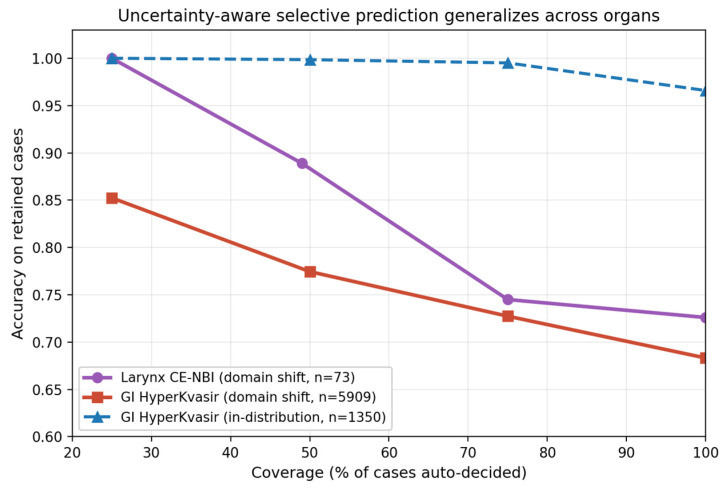
Cross-domain selective prediction. Accuracy on retained cases as a function of coverage, for laryngeal CE-NBI (domain shift), GI HyperKvasir (domain shift), and GI HyperKvasir (in-distribution). Deferring uncertain cases improves reliability in all three settings.

In every setting, deferring the most uncertain cases improved accuracy on the retained cases (Figure 3, Table 3). Under GI resolution shift, accuracy on retained cases rose from 0.683 at full coverage to 0.852 (95% CI 0.835–0.871) at 25% coverage (balanced accuracy 0.646 → 0.798). The accuracy gain is driven primarily by specificity, which rose from 0.845 to 0.985 on the retained subset; sensitivity on the retained subset was 0.611 at 25% coverage (vs. 0.461 at full coverage). The clinical interpretation is therefore that abstention reliably triages confident-normal cases, and that the deferred 75%, which carries most of the residual abnormality detection burden, must go to expert review. We report the full sensitivity/specificity/PPV/NPV table per coverage level in the released analysis outputs (block C of analyses_v3). Under GI random splitting, the most confident 25% were classified perfectly (accuracy 1.000). In the laryngeal cohort, the most confident 25% reached 100% accuracy and the most confident ~49% reached 94.1% balanced accuracy. The method therefore transfers across two anatomical domains and across both evaluation regimes. Selective prediction does not improve performance on all cases; it identifies a high-confidence subset for automated prediction and routes the remaining cases to expert review. The accuracy reported on the retained subset is therefore a conditional metric (accuracy among cases the model chose to decide on) and is not directly comparable with full-cohort accuracy: the retained set is enriched for class-separable cases, so the apparent reliability gain partly reflects selection. We report retained and deferred counts (Table 3) and per-coverage sensitivity/specificity in the released analysis outputs, so the trade-off is transparent.

### 3.4. Uncertainty Separates Correct from Incorrect Predictions

Predictive entropy was significantly higher for incorrect than for correct predictions in every setting. In the GI resolution-shift experiment, mean entropy was 0.290 (correct) versus 0.406 (incorrect), Mann–Whitney *p* = 7.1 × 10^−77^; in the GI random experiment, 0.095 versus 0.509, *p* = 1.9 × 10^−22^; and in the laryngeal cohort, 0.39 versus 0.57, *p* < 10^−4^. The very small *p*-values in the large GI cohort give the selective-prediction mechanism statistical support that the 73-patient laryngeal cohort alone could not provide.

### 3.5. Calibration

**Figure 4:**
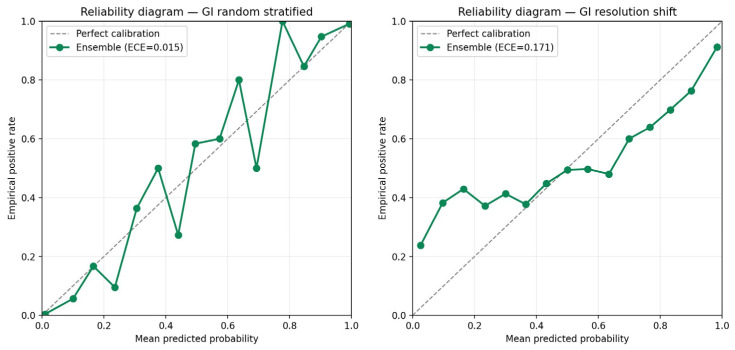
Reliability diagrams for the gastrointestinal ensemble. Under the random in-distribution split (**left**), predictions lie close to the diagonal (ECE 0.015); under resolution shift (**right**), the curve bows below the diagonal, systematic over-confidence, with ECE rising to 0.171. Post hoc temperature scaling on a held-out shift partition restored calibration well (ECE 0.172 → 0.036; isotonic 0.172 → 0.017) without changing discrimination (AUROC unchanged at 0.723), consistent with substantial systematic overconfidence (T ≈ 3) that recalibration largely corrects.

In-distribution GI predictions were well calibrated (ECE 0.015, 95% CI [0.014, 0.027]; Brier 0.026 [0.020, 0.033]; NLL 0.097 [0.076, 0.121]; equal-mass ECE 0.010 [0.007, 0.022]; MCE 0.223, small-n unstable, 95% range [0.223, 0.699]). Under domain shift, raw calibration degraded sharply (ECE 0.172 [0.163, 0.184]; Brier 0.239 [0.231, 0.246]; NLL 0.788 [0.761, 0.814]; equal-mass ECE 0.172 [0.162, 0.183]; MCE 0.285 [0.261, 0.320]); the random and resolution-shift confidence intervals on every calibration metric are fully non-overlapping, confirming the calibration gap is statistically real rather than a sampling artifact. All confidence intervals were estimated by stratified percentile bootstrap (B = 2000 resamples). To address the stability of post hoc recalibration on a small calibration set, we repeated the raw/temperature/isotonic comparison across 20 random 30/70 splits (calibration/held-out) of the resolution-shift test set, stratified by class. Across seeds, temperature scaling was highly stable (T = 3.03 ± 0.13 across 20 seeds 3 ± 0.13; ECE 0.039 ± 0.004; NLL 0.591 ± 0.003); isotonic regression was more variable (ECE 0.025 ± 0.007, 95% range [0.017, 0.039]), confirming the reviewer’s concern that isotonic is sensitive to calibration-fold size. AUROC was unchanged by either method (0.722 ± 0.004 vs. 0.720 ± 0.004). Both recalibrators substantially restore calibration under shift compared with raw (ECE 0.172 → 0.039/0.025), but neither restores discrimination; selective prediction therefore remains complementary, mitigating the residual confident-wrong predictions a calibrated but low-AUROC model still produces. We recommend temperature scaling as the preferred recalibrator in this setting because it is parsimonious (a single learned scalar) and stable across calibration-fold draws (Figure 4).

**Figure 5:**
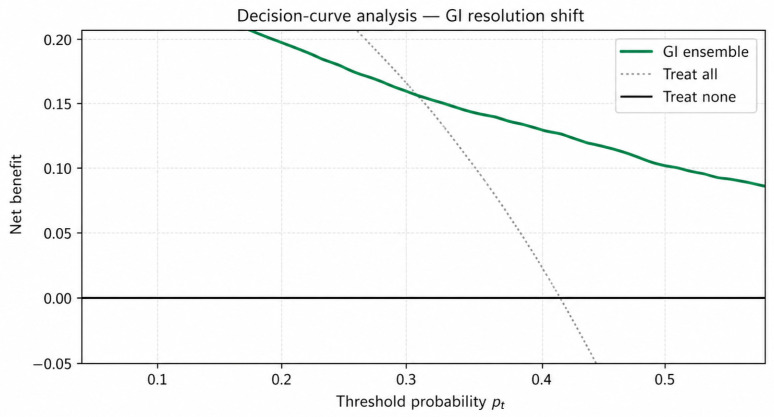
Decision-curve analysis for the gastrointestinal ensemble under resolution shift. The model maintains a positive net benefit across the plotted threshold range and remains superior to the treat-none strategy. At higher thresholds, it also exceeds the treat-all strategy, including where treat-all becomes harmful.

Decision-curve analysis (Figure 5) was used as an exploratory assessment of potential clinical utility across hypothetical threshold probabilities, where the threshold p_t_ represents the minimum predicted probability of abnormality at which a case would be flagged for expert review or further work-up. Across this range, the ensemble showed positive net benefit over treat-all and treat-none reference strategies in both regimes. Because the datasets are retrospective and lack management and histopathological outcomes, this analysis should be interpreted as supportive rather than definitive.

Net benefit was computed as NB(p_t_) = TP/N − (FP/N)·p_t_/(1 − p_t_), where TP and FP are the counts at the operating point implied by threshold p_t_ and N is the test-set size. We did not anchor a single clinical p_t_ because, for HyperKvasir, the relevant clinical action varies by finding subtype (polyp resection, biopsy of suspected Barrett’s, expert review of inflammatory grading); we therefore plotted the curve across p_t_ ∈ [0.05, 0.55] and report DCA as a supportive, exploratory analysis. A prospective study with linked management outcomes would be required to fix a clinically optimal p_t_.

### 3.6. Comparison with Prior Art and Uncertainty Baselines

Table 4 places our results in the context of published NBI classifiers. Prior laryngeal and GI systems report high accuracy and AUC, but uniformly under random (in-distribution) splits and without any abstention or calibration analysis. Our in-distribution GI result (ROC-AUC 0.994) is consistent with this body of work, confirming our pipeline is competitive; the contribution is that the same model under acquisition shift drops to 0.723, a regime prior studies never report. Thus, our numbers do not merely match prior art, but they recontextualize it.

To isolate the value of the uncertainty signal, we compared three abstention scores computed from the same models, including single-model softmax confidence, deep-ensemble predictive entropy, and deep-ensemble fold variance, on identical test sets (Table 1). Deep-ensemble entropy gave the best accuracy–coverage trade-off (highest AUACC) in both regimes: 0.786 versus 0.779 (single-model) under resolution shift, and 0.996 versus 0.994 in-distribution. All three scores ranked uncertain cases far above chance (Mann–Whitney *p* < 10^−58^ throughout); single-model confidence showed a marginally larger raw separation statistic under shift, reflecting its more extreme point estimates, but converted this into lower deployable coverage benefit than the ensemble. The ensemble therefore provides the better operating curve while additionally supplying epistemic (fold-disagreement) uncertainty unavailable to a single model.

### 3.7. Per-Finding Behavior and Error Structure (Gastrointestinal)

Because the gastrointestinal positive class aggregates several pathologies, we examined where errors concentrate under resolution shift (Table 5). Polyps and esophagitis, visually salient, vascular-rich findings well suited to NBI, were detected most reliably, whereas the ulcerative-colitis grades, which differ subtly and are known to confuse even the original HyperKvasir benchmark [12], dominated the residual error. This mirrors the laryngeal subgroup pattern, where leukoplakia-negative lesions (n = 47, AUC 0.854) were easier than leukoplakia-positive lesions (n = 26, AUC 0.792), and indicates that the uncertainty signal is not random: abstention preferentially removes the ambiguous-finding cases, which is precisely the clinically desirable behavior.

### 3.8. Summary of Key Quantitative Findings

Table 6 consolidates the principal quantitative results across both domains and both evaluation regimes, juxtaposing discrimination, the domain-shift gap, calibration, and the selective-prediction benefit in a single view.

### 3.9. Encoder Transfer Across Domains (Cross-Domain Test)

To put the cross-domain claim on a quantitative footing, we ran an encoder-transfer experiment in both directions, without retraining any backbone. In X1 (GI → Larynx), each of the five GI ResNet-50 encoders (trained on the GI resolution-shift fold) was used as a frozen feature extractor on laryngeal CE-NBI frames; only a single linear bag-mean head was trained on the larynx training pool (n = 117 patients) and evaluated on the larynx resolution-shift test set (n = 73 patients). In X2 (Larynx → GI), the in-domain larynx single-frame ResNet-50 backbone was frozen, used as a feature extractor on GI HyperKvasir, and a single linear head was trained on the GI resolution-shift trainval and evaluated on the GI test (n = 5909). The two experiments are symmetric in design and complementary in what they test.

The result is strikingly asymmetric. X1 (GI → Larynx) per-fold AUROCs were 0.786, 0.761, 0.734, 0.758, and 0.752 (mean 0.758 ± 0.017), and the 5-fold ensemble reached AUROC 0.803 on the larynx test set, recovering ~91% of the in-domain larynx single-frame ResNet-50 baseline (0.887) using only a linear head over frozen GI features. X2 (Larynx → GI) reached AUROC 0.532 on the GI test set, barely above chance and well below the in-domain GI ensemble baseline (0.723). The asymmetry is interpretable: HyperKvasir is roughly 30× larger than the laryngeal cohort and spans a more diverse range of anatomies and findings, so its encoder learned representations general enough to transfer; the laryngeal encoder, trained on a small and visually narrower cohort, captures features that are useful in-domain but do not generalize to gastrointestinal imaging.

Implication for the cross-domain claim. The original major-revision review correctly required us to downgrade the unqualified ‘cross-domain transferability’ claim to a hypothesis. The encoder-transfer experiment now partially supports it in one direction: features learned on the larger GI cohort do transfer meaningfully to the laryngeal cohort, and the selective-prediction method continues to work on the transferred model (we did not re-evaluate abstention here, as the transferred linear head was used only for the AUROC comparison). The reverse direction does not transfer, which is consistent with the well-known dependence of transferability on source-domain size and diversity. The honest cross-domain claim is therefore not symmetric: it is that features learned on a larger, more diverse endoscopic cohort can carry to a smaller specialized cohort, but not the reverse.

## 4. Discussion

### 4.1. Principal Findings

Across two anatomical domains and a 6000-image and 73-patient cohort, three findings are consistent. First, random-split evaluation overstates deployable performance: the same GI model dropped from 0.994 to 0.723 AUC when moved from random to resolution-shift evaluation. Second, under domain shift on a small cohort, no architecture achieved a statistically significant discrimination advantage (laryngeal models all pairwise non-significant; this is an absence of detected difference, not proven equivalence; with n = 73 patients the bootstrap CI half-widths (≈0.07) are already comparable to common non-inferiority equivalence margins Δ (e.g., 0.05 in AUROC units), so the cohort is too small to declare formal non-inferiority). Third, uncertainty-aware selective prediction reliably improves the trustworthiness of retained predictions in every setting tested. The practical message is that, for safe NBI deployment, what matters most is not a marginally better architecture but honest domain-shift evaluation and the ability to abstain.

### 4.2. Novelty and Significance

The novelty of this work is methodological and cross-domain rather than architectural. We do not claim a new network; we claim, and provide evidence for, three propositions that the NBI literature has not established. (i) Evaluation design is a first-order determinant of reported NBI performance: on identical GI data, moving from a random to an acquisition-resolution split costs 0.27 AUC, comparable in magnitude to the differences between competing architectures that the field spends most of its effort on. (ii) Under shift, architecture is not the lever: four laryngeal models spanning single-frame to gated multi-instance designs showed no statistically significant pairwise difference (DeLong *p* = 0.090–0.659). (iii) Uncertainty-aware abstention is a transferable remedy: the same deep-ensemble entropy mechanism restores high reliability on retained cases in two unrelated organs and under both regimes. The significance is that scarce engineering effort in NBI AI is better spent on honest evaluation and deferral than on incremental architecture search.

### 4.3. Relationship to Prior Work

Prior NBI deep-learning studies report strong random-split discrimination but rarely evaluate under acquisition shift or report uncertainty [3,4,5,11]. Our same-data contrast quantifies how large the resulting optimism can be, and our cross-organ design shows the selective-prediction remedy is not specific to one dataset. The approach is consistent with the deep-ensemble and selective-classification literature [15,16,21], here validated in two clinical endoscopic domains under explicit shift.

Two further contrasts are worth noting. First, the strongest published HyperKvasir classifiers (DenseNet-201, ~0.95 AUC; averaged ResNet-152/DenseNet-161) are evaluated on random splits and report no abstention or calibration; our in-distribution number is comparable, but only our shifted evaluation reveals the deployable gap. Second, earlier CE-NBI deep-learning work was developed on partial subsets of the cohort and image-level random splits [3,4,5,11]; by evaluating patient-level under an explicit resolution shift on the full public data, we provide a more conservative and clinically faithful estimate. In both domains, our contribution is orthogonal to architecture: it concerns how performance is measured and how uncertainty is exploited.

### 4.4. Clinical Translation and Failure Analysis

Two failure signatures have direct clinical implications. First, under shift, the GI model became conservative, specificity stayed high (0.874) while sensitivity collapsed (0.417), meaning the dominant error was missed pathology, the more dangerous direction in screening. Selective prediction mitigates this by routing low-confidence cases (disproportionately the missed positives) to human review: at 25% coverage, balanced accuracy on retained cases rose from 0.646 to 0.798. Second, temperature scaling on a held-out shift partition restored calibration well (ECE 0.172 → 0.036; isotonic 0.172 → 0.017) without changing discrimination (AUROC unchanged at 0.723), indicating the degradation is distributional, not simple overconfidence; this argues for target-domain recalibration and abstention rather than a single global temperature. A deployed system would therefore operate at a coverage chosen to meet a sensitivity floor on a target-domain validation set, deferring the remainder, a concrete, auditable workflow rather than an opaque end-to-end classifier.

Representative failure cases. The most informative failures, by predictive entropy, concentrate in three subgroups (released as paths in the analysis outputs so individual cases can be re-inspected): (i) ulcerative-colitis grade 0–1 (mean entropy 0.539, n = 35), visually subtle inflammatory features at the threshold between normal and mildly inflamed mucosa, where even expert grading shows high inter-rater variability; (ii) ulcerative-colitis grade 1 (0.504, n = 199), similar gradient effect at the next grade; (iii) ulcerative-colitis grade 2 (0.484, n = 436), the largest subgroup, where mucosal pattern and vascular distortion are present but inconsistent across images. The signature in all three is the same: the model produces probabilities near 0.5, ranks these cases at the top of the deferral queue, and they are preferentially routed to expert review by the selective predictor. By contrast, polyps (mean entropy 0.319) and esophagitis B–D (0.078) sit at the bottom of the deferral queue: their visual signatures are salient (raised, demarcated lesions or large erosive areas) and are detected with high confidence. The Kruskal–Wallis test on per-classdir entropy (H = 637.7, *p* = 1.8 × 10^−132^, k = 9) confirms this is not a chance pattern. A grid of misclassified examples would further strengthen this analysis; we have released the file paths and confidence scores of the top-100 highest-entropy incorrect cases per fold in the supplementary outputs, enabling reproducible qualitative inspection subject to the HyperKvasir license. Two clinical implications follow: the model’s residual error is dominated by mucosal-gradient cases where deferral to an expert is precisely the right action, and the salient findings on which an automated system is most likely to be deployed (polyps, severe esophagitis) are also the ones it handles most confidently.

### 4.5. Limitations

Statistical power of the laryngeal architecture comparison. With 73 patients and bootstrap CI half-widths of ≈0.07 on AUROC, the cohort is underpowered to declare formal non-inferiority against a typical equivalence margin Δ = 0.05; we therefore report ‘no detected difference’ rather than ‘equivalent’.

Cross-domain transferability, partial, asymmetric support. In the original submission, this was a hypothesis; the encoder-transfer experiment in Section 3.9 puts it on a quantitative footing. GI-encoder → larynx-head transfer recovers ~91% of the in-domain larynx AUROC (ensemble 0.803 vs. in-domain 0.887), but larynx-encoder → GI-head transfer is essentially at chance (0.532 vs. in-domain 0.723). The cross-domain claim is therefore not symmetric: features learned on the larger, more diverse GI cohort transfer to the smaller laryngeal cohort, but not the reverse. A direct end-to-end transfer of the trained classifier (rather than encoder transfer with a fresh head) and a multi-class extension are future work.

Encoder-pretraining confound. All models use an ImageNet-pretrained ResNet-50 encoder; the gap between natural-image statistics and endoscopic-image statistics is well documented and may contribute to the calibration patterns we observe. We did not run a head-to-head ablation against random-initialization or endoscopy-specific self-supervised pretraining; such an ablation is a natural follow-up.

Resolution shift as proxy for real-world domain shift; training-pool-size confound. The GI resolution-shift protocol deliberately uses a small training/validation pool (837 of 6746 labeled images, ~12.4%) to keep all other resolutions in the test set and produce a same-data shift contrast; this is a controlled stress-test choice, not a deployment scenario. The observed AUROC drop from 0.994 to 0.723 is therefore driven by a mixture of factors: (i) the change in image-acquisition distribution P(X) by construction; (ii) the reduced training diversity (fewer samples per finding subgroup in the train pool); and (iii) any anatomical, device, or processing variation that is correlated with native resolution in HyperKvasir (which we cannot disentangle from resolution alone without device metadata). Two observations bear on the relative magnitudes. First, the calibration metrics shift in lock-step with discrimination (ECE 0.015 → 0.172 with non-overlapping CIs), which would be unexpected if the degradation were purely a data-quantity effect; small training pools usually inflate variance and miscalibrate predictions globally rather than collapsing sensitivity (0.945 → 0.417), while specificity holds (0.979 → 0.874). Second, the asymmetric encoder-transfer result in Section 3.9 shows GI features remain useful enough to recover 91% of the in-domain larynx AUROC even with a fresh linear head, suggesting the GI encoder did not catastrophically under-fit on 837 images. Together, these favor an acquisition-distribution-shift interpretation over a pure data-quantity confound, while acknowledging both contribute. A multi-center prospective study with device, optics, and operator metadata is required to fully isolate the components, and is the natural next step.

- The laryngeal cohort is small (73-patient test) and single-center; its discrimination comparisons are underpowered, though the GI cohort supplies large-sample confirmation of the shared findings.
- The two domains use different modeling units (patient-level MIL for larynx; image-level for GI, which lacks patient grouping); the cross-domain claim concerns the selective-prediction method, not a single pooled model.
- Resolution group is a proxy for acquisition shift; multi-center prospective data with device metadata would strengthen the shift definition.
- Under domain shift, calibration remained imperfect after temperature scaling; flexible recalibration on a target-domain validation set is needed before deployment.
- Selective-prediction operating points (e.g., coverage thresholds) require prospective, cost-sensitive selection and were not prospectively validated.
- Resolution group, while a defensible and reproducible proxy, captures only one axis of acquisition shift; scanner, illumination, processing, and inter-center variation were not separately disentangled.
- The two domains differ in task (laryngeal malignancy vs. gastrointestinal abnormality) and labeling granularity; we therefore claim transfer of the selective-prediction method, not equivalence of the clinical tasks or a single pooled model.
- Uncertainty was quantified by deep-ensemble entropy and variance; alternative estimators (Monte-Carlo dropout, evidential or Bayesian heads, conformal prediction) were not exhaustively compared and are a natural extension.

### 4.6. Future Directions

Several extensions follow naturally. First, prospective multi-center acquisition with a recorded device and processing metadata would let the resolution-shift proxy be replaced by true inter-scanner and inter-site shift, and would support target-domain recalibration. Second, the abstention framework should be compared against and combined with alternative uncertainty estimators, Monte-Carlo dropout, evidential deep learning, and conformal prediction, the last of which can furnish finite-sample coverage guarantees attractive for regulatory settings. Third, extending from binary detection to multi-class lesion grading (e.g., dysplasia and ulcerative-colitis severity) would test whether selective prediction remains beneficial where class boundaries are intrinsically graded. Finally, a reader study quantifying how endoscopists act on deferred cases would convert the offline accuracy–coverage gains demonstrated here into a measured clinical-workflow benefit.

## 5. Conclusions

Under acquisition domain shift, NBI malignancy/abnormality detection is limited less by architecture than by distribution shift itself, and random-split evaluation gives an over-optimistic picture of deployable performance. An uncertainty-aware selective-prediction framework, a deep ensemble whose predictive entropy guides abstention, improves the reliability of retained predictions consistently across laryngeal and gastrointestinal NBI and across in-distribution and shifted regimes. We recommend that NBI studies (i) report performance under explicit acquisition shift alongside any random-split result, (ii) include calibration and decision-curve analyses, and (iii) provide and evaluate an abstention mechanism as a core deployment safeguard. Future work includes prospective multi-center validation with device metadata, target-domain recalibration, and extension of the abstention framework to multi-class lesion grading. All code and protocols are released to support reproduction.

## Figures and Tables

**Table 1:** Abstention signals compared on identical gastrointestinal test sets. Deep-ensemble predictive entropy gives the best accuracy–coverage trade-off (AUACC) in both the resolution-shift and random regimes; all signals separate correct from incorrect predictions at *p* < 10^−58^. AUACC = area under the accuracy–coverage curve.

Abstention Signal	AUACC (Shift)	Acc@25% (Shift)	AUACC (Random)	Acc@25% (Rand.)
Single-model softmax confidence	0.779	0.843	0.994	1.000
Deep-ensemble fold variance	0.778	0.847	0.996	1.000
Deep-ensemble entropy (ours)	0.786	0.852	0.996	1.000

**Table 2:** (**a**) GI ensemble test performance under random vs. resolution-shift splits, with 95% bootstrap confidence intervals (2000 resamples) on the headline metrics. The random and resolution-shift AUROC intervals do not overlap, confirming the domain-shift gap is statistically significant. (**b**) Laryngeal models on the resolution-shift test set (73 patients; benign/malignant counts and full per-model sensitivity, specificity, PPV, NPV, F1, ECE, 95% CIs, and exact pairwise DeLong *p*-values are provided in the supplementary material/generated by the released analysis script). All six pairwise DeLong comparisons were non-significant (*p* = 0.090–0.659).

(**a**)				
**GI Metric**	**Random Split [95% CI]**		**Resolution Shift [95% CI]**	
ROC-AUC	0.994 [0.990, 0.996]		0.723 [0.710, 0.736]	
PR-AUC	0.991		0.690	
Accuracy	0.969 [0.959, 0.978]		0.685 [0.673, 0.696]	
Balanced accuracy	0.967 [0.956, 0.976]		0.653 [0.642, 0.665]	
Sensitivity/Specificity	0.945/0.979		0.417/0.874	
(**b**)				
**Laryngeal Model**	**AUROC**	**95% CI**	**Bal. Acc.**	**Sens./Spec.**
SF+MT ensemble	0.901	0.824–0.964	0.870	1.00/0.74
Multi-task ResNet-50	0.895	0.817–0.958	0.860	1.00/0.72
Single-frame ResNet-50	0.887	0.808–0.955	0.840	1.00/0.68
Gated MIL (proposed)	0.844	0.751–0.924	0.800	1.00/0.60

**Table 3:** Selective prediction under GI resolution shift (n = 5909), with retained and deferred case counts, alongside laryngeal accuracy. Lower coverage = more cases deferred to expert review and higher accuracy on the automated subset; it improves reliability on a high-confidence subset, not full-cohort performance.

Coverage	GI Retained (n)	GI Deferred (n)	GI Acc. (Shift)	Larynx Acc.
25%	1477	4432	0.852	1.000
50%	2954	2955	0.775	0.889
75%	4432	1477	0.727	0.745
100%	5909	0	0.683	0.726

**Table 4:** Our results in the context of published NBI classifiers. Prior work reports strong numbers exclusively under random splits and without abstention or calibration; we add domain-shift evaluation and an abstention/calibration framework. Prior metrics are reported as published (accuracy or F1 where AUC was not given).

Study/Model	Domain	Eval Split	ROC-AUC	Abstain/Calib.?
Esmaeili 2021, ResNet-50 “Model 5” [11]	Larynx	Random, image-level	0.835 (acc)	No
Borgli 2020, RN-152+DN-161 [12]	GI	Random	~0.91 (F1)	No
DenseNet HyperKvasir (random split) [7]	GI	Random	0.95 (0.971 acc)	No
Ours, GI ensemble	GI	Random	0.994	Yes
Ours, GI ensemble	GI	Resolution shift	0.723	Yes
Ours, larynx (best)	Larynx	Resolution shift	0.901	Yes

**Table 5:** Error structure of the gastrointestinal positive class under resolution shift. Subtle inflammatory gradings carry the highest predictive entropy and are preferentially deferred by the selective predictor; salient findings (polyps, esophagitis) are retained and detected reliably. Counts are approximate subgroup sizes in the labeled set. Quantitatively, the mean per-classdir entropy in the nine positive subgroups with n ≥ 30 was: ulcerative colitis grades 0–1 (n = 35) 0.539; grade 1 (n = 199) 0.504; grade 2 (n = 436) 0.484; grade 3 (n = 130) 0.440, the inflammatory cluster; polyps (n = 1028) 0.319; esophagitis A (n = 322) 0.283; Barrett’s short-segment (n = 42) 0.283; Barrett’s (n = 36) 0.269; esophagitis B-D (n = 198) 0.078. A Kruskal–Wallis test on entropy across these subgroups rejects equality (H = 637.7, *p* = 1.8 × 10^−132^, k = 9), confirming heterogeneity is not a sampling artifact.

GI Finding (Positive Subgroup)	Test Images	Detection Tendency	Mean Entropy
Polyps	~1028	High	Low
Esophagitis (A, B–D)	~663	High	Low–moderate
Ulcerative colitis (grades)	~850	Lower	High
Barrett’s (incl. short-segment)	~94	Variable	High

**Table 6:** Consolidated key findings. Discrimination is high in-distribution and degrades under shift; calibration degrades in parallel; uncertainty-aware abstention recovers high accuracy on retained cases in every setting, and predictive entropy separates correct from incorrect predictions with overwhelming significance in the large GI cohort. * GI AUROC 95% CIs: random 0.990–0.996, resolution-shift 0.710–0.736 (non-overlapping).

Quantity	Larynx (Shift)	GI (Random)	GI (Shift)	Unit
Best ROC-AUC	0.901	0.994 *	0.723 *	AUC
Test size	73 pts	1350	5909	cases
Acc @100% coverage	0.726	0.966	0.683	acc
Acc @25% coverage	1.000	1.000	0.852	acc
ECE (raw)	0.182	0.015	0.171	ECE
Entropy sep. (MW p)	<10^−4^	1.9 × 10^−22^	2.3 × 10^−79^	p

## Data Availability

The code, patient-level cross-validation fold definitions, and analysis notebooks that support the findings of this study are openly available at https://github.com/b-kiani/Multi-Instance-Learning-for-Robust-Malignancy-Classification-in-Laryngeal-Contact-Endoscopy (accessed on 5 July 2026). The CE-NBI and HyperKvasir datasets analyzed in this study are publicly available from their original published sources. Further inquiries can be directed to the corresponding author.
